# The orexinergic system influences conditioned odor aversion learning in the rat: a theory on the processes and hypothesis on the circuit involved

**DOI:** 10.3389/fnbeh.2014.00164

**Published:** 2014-05-06

**Authors:** Barbara Ferry

**Affiliations:** Centre of Research in Neuroscience Lyon, CNRS UMR 5292 - INSERM U1028 UCBL1Lyon, France

**Keywords:** associative learning, olfactory memory, orexin, fasting, rat

## Abstract

A large variety of behaviors that are essential for animal survival depend on the perception and processing of surrounding smells present in the natural environment. In particular, food-search behavior, which is conditioned by hunger, is directly driven by the perception of odors associated with food, and feeding status modulates olfactory sensitivity. The orexinergic hypothalamic peptide orexin A (OXA), one of the central and peripheral hormones that triggers food intake, has been shown to increase olfactory sensitivity in various experimental conditions including the conditioned odor aversion learning paradigm (COA). COA is an associative task that corresponds to the association between an olfactory conditioned stimulus (CS) and a delayed gastric malaise. Previous studies have shown that this association is formed only if the delay separating the CS presentation from the malaise is short, suggesting that the memory trace of the odor is relatively unstable. To test the selectivity of the OXA system in olfactory sensitivity, a recent study compared the effects of fasting and of central infusion of OXA during the acquisition of COA. Results showed that the increased olfactory sensitivity induced by fasting and by OXA infusion was accompanied by enhanced COA learning performances. In reference to the duration of action of OXA, the present work details the results obtained during the successive COA extinction tests and suggests a hypothesis concerning the role of the OXA component of fasting on the memory processes underlying CS-malaise association during COA. Moreover, referring to previous data in the literature we suggest a functional circuit model where fasting modulates olfactory memory processes through direct and/or indirect activation of particular OXA brain targets including the olfactory bulb, the locus coeruleus (LC) and the amygdala.

## Introduction

A large variety of behaviors that are essential for animal survival depend on the sensory perception and processing of odors present in the natural environment. In particular, food-search behavior, which is conditioned by hunger, is directly driven by the perception of odors associated with food (Le Magnen, 1959) and several studies have demonstrated that odor processing is influenced by the nutritional status of the animal. For example, olfactory system activity was shown to be directly modulated according to hunger and satiation status (Pager et al., 1972; Pager, 1974, 1978; Royet et al., 1983; Apelbaum et al., 2005). Moreover, fasting enhanced odor detection in rats whereas satiety reduced detection of odors in general (Aimé et al., 2007), and more precisely of one odorant specifically associated with the food type involved in the satiation (O’Doherty et al., 2000; Mulligan et al., 2002). Some data suggest that the central nervous system (CNS) regulates food-search behavior by modulating the detection threshold of the food odorant itself through centrifugal innervations (Doucette et al., 2007; Doucette and Restrepo, 2008; Fletcher and Chen, 2010). A large body of data indicates that the hypothalamus plays an important role in this process. Firstly, anatomic characterization of the lateral hypothalamus (LH) has shown the existence of a functional loop between structures involved in the first level of odor detection and the hypothalamus (Peyron et al., 1998; Sakurai, 2005; Swanson et al., 2005; Hahn and Swanson, 2010). Secondly, the crucial role of the basal hypothalamus, and in particular of orexigenic (appetite-stimulating) and anorexigenic (appetite-inhibiting) neurochemicals, in appetite regulation and energy balance has long been established (see Rodgers et al., 2002 for review). Thirdly, among the multitude of neurochemicals found in the hypothalamus, the most recently discovered orexigenic peptides (orexin A, OXA and orexin B, OXB) have been shown to be strongly involved in the regulation of feeding and energy metabolism (see Willie et al., 2001 for review) and the OXA has been involved in olfactory sensitivity. In particular, our team showed that intracerebroventricular (icv) infusion of OXA in rat increased olfactory detection performance in the same way as physiologically induced fasting (Aimé et al., 2007; Julliard et al., 2007). Fourthly, lateral hypothalamic orexin neurons project directly to the olfactory bulb (OB; Peyron et al., 1998; Nambu et al., 1999; Caillol et al., 2003; Shibata et al., 2008) and the two classes of receptors for OXA and for OXB have been found in OB neurons (Caillol et al., 2003; Hardy et al., 2005). In addition, central infusion of OXA increased OB Fos responses to food odor in both fasted and satiated animals (Prud’homme et al., 2009). All these results indicate that the orexin system is involved in the control of feeding behavior by modulating olfactory sensitivity.

It is, however, very unlikely that olfactory sensitivity is completely dissociated from olfactory memory. Rusiniak et al. (1982) and Slotnick et al. (1997) showed that the more intense the odor, the stronger the memory of its association with a reinforcement. Interestingly, other than its projection on the primary olfactory centers, the hypothalamic OXA neurons project to various structures involved in olfactory associative learning (see Rodgers et al., 2002 for review). Moreover, the OXA system was shown to be involved in the memory processes underlying various kinds of learning (Jaeger et al., 2002; Telegdy and Adamik, 2002; Mair and Hembrook, 2008; Di Sebastiano et al., 2011; Sears et al., 2013; Soya et al., 2013). In addition, Touzani and Sclafani (2002) have shown that the lesion of the LH induced a deficit in conditioned flavor aversion paradigms. Therefore, it can be suggested that the OXA system, may influence odor memory formation, directly, by modulating olfactory sensitivity, and indirectly, by activating particular hypothalamus target regions through the modulation of olfactory sensitivity.

In a natural environment, the relevance of the odor coming from a food source encountered by an animal during food-search is a crucial key, determining approach and ingestion of the food. Whether the odor of the food is new to the animal or has previously acquired a hedonic valence during a first intake experience will condition either approach or avoidance. Acquisition of hedonic valence by a food item has been shown to result from conditioned learning during which the sensory stimuli characterizing a particular food (odor and taste) become associated with the positive (energy input) or negative (gastric malaise, poisoning) consequences of the ingestion of the food, so that processing the odor and taste stimuli will cue the appropriate approach or avoidance responses (Rescorla, 1988; Holland, 1990; Mackintosh, 1991). These kinds of association have been experimentally studied for years (Slotnick and Katz, 1974; Nigrosh et al., 1975; Slotnick, 1984) and conditioned food aversion paradigms, such as conditioned taste or odor/taste-potentiated odor aversion learning, have provided fundamental insights into the mechanisms and CNS structures involved in food-reward/food-poisoning associations (see Miranda, 2012 for review).

One such paradigm, conditioned odor aversion (COA), is the avoidance of an odorized-tasteless solution (conditioned stimulus, CS) the ingestion of which precedes toxicosis (unconditioned stimulus, US). During COA acquisition, the presentation of the CS is separated from the US administration by a temporal gap that can be of various amplitudes depending on whether the CS is mixed (proximal presentation; Slotnick et al., 1997; Chapuis et al., 2007) or presented close to the solution (Palmerino et al., 1980; Rusiniak et al., 1982; Ferry et al., 1996, 2006). Thus COA is a trace conditioning that has been suggested to result from the association of the memory trace of the CS and the delayed US (see Bures and Buresova, 1990; Roldan and Bures, 1994).

Taking these data together with the fact that (i) the amygdala has been widely involved in the processes underlying the association between the CS and the delayed US during COA (see Miranda, 2012 for review), (ii) OXA neurons project to the amygdala and (iii) hypothalamic OXA neurons are activated by cues associated with consummatory rewards such as food (Harris et al., 2005), it is suggested that the OXA system may play a role in the learning and memory processes linked to higher cognitive aspects of feeding.

In order to test this hypothesis, a recent study has aimed to describe the role of the central OXA system in COA learning (Ferry and Duchamp-Viret, 2014). The results showed that fasting and icv infusion of OXA before the acquisition significantly enhanced COA performances. Moreover in that study, data obtained during the elevated plus maze task showed enhanced anxiety in Fasted but not in OXA infused animals suggesting that the enhancing effect of fasting on COA performances is likely mediated, at least in part, by the OXA component of fasting. In order to further precise which process the OXA system is involved in, the present experiment extends our previous one (Ferry and Duchamp-Viret, 2014) and includes a comparison between the effect of icv OXA infusion and fasting on the extinction of COA learning.

## Material and Methods

The description of the material and methods has been simplified and subjects, surgery and microinfusion procedure are detailed in a previous article (Ferry and Duchamp-Viret, 2014).

Briefly, three groups of animals were acclimated for 7 days to a 23 h 45 min water deprivation schedule. Groups OXA and artificial CSF (aCSF) were constituted by animals implanted in the lateral ventricle that were microinfused with OXA (10 µg dissolved in 3 µl of sterile CSF, Sigma) or aCSF (3 µl, Harvard Apparatus) 20 min before the acquisition of the COA task (on Day 8). A third experimental group (Fasted) was constituted by animals placed in a 24-h food-deprivation schedule before acquisition of COA. Acquisition of COA consisted by the presentation of an olfactory CS (scented water corresponding to isoamylacetate, Sigma-Aldrich France, mixed with tap water at a final concentration of 10^−6^) followed 20 min after by an injection of 0.15 M lithium chloride-inducing gastric malaise (LiCl, 10 ml/kg; i.p., US). Conditioned aversion to the odor was tested 48 h later (on Day 11) and COA extinction learning took place from Days 12 to 15. All the testing and extinction sessions were conducted under a food-satiated condition in order to prevent any effect of fasting on the olfactory sensitivity between groups (Aimé et al., 2007; Julliard et al., 2007) and consisted of 15-min presentation of the CS (one bottle test). This procedure is based on previous studies (Julliard et al., 2007 for the OXA infusion and fasting Ferry et al., 2007 for the COA procedure). In order to focus our study on the role of OXA system in memory processes underlying COA learning, it is important to note that animals in our procedure received one OXA infusion before the CS-US pairing and not during the test.

## Results

**Figure 1:**
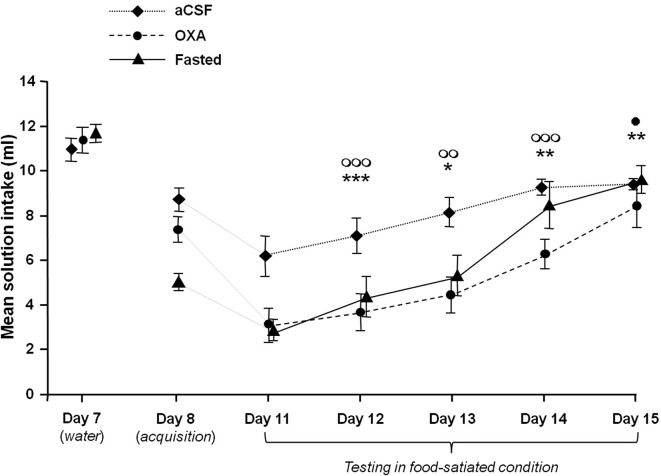
**Effects of icv infusion of orexin (10 µg/3 µl, OXA group), icv infusion of artificial CSF (3 µl, aCSF group) and food-deprivation (Fasted group) on COA acquisition and extinction**. The curves represent mean odorized-solution intakes (± SEM) for each group from Day 8 (acquisition) to Day 15. The mean water intake measured on the last day of habituation (Day 7) is also represented for each group for purposes of comparison. The Fasted group was food-deprived only during acquisition. All animals were habituated and tested while food satiated. *, ** and ***: *P* < 0.05, *P* < 0.01 and *p* < 0.001 between OXA and aCSF group. ◯◯ and ◯◯◯ *P* < 0.01 and 0.001 between Fasted and aCSF group. • *P* < 0.05 between OXA and Fasted group.

Figure 1 illustrates the mean scented water intake measured during the acquisition (Day 8), the test (Day 11) and the four successive COA extinction sessions (Days 11 to 15) for the various groups. For comparison purpose, the last water-drinking habituation session (Day 7) has also been represented. As shown by this figure, performances obtained during the test and the number of extinction sessions differed between groups, depending on the treatment.

A two-factor ANOVA with repeated measures with Treatment (aCSF, OXA and Fasted) as between-subjects variable and Session of extinction (Day 11 to Day 15) as within-subject variable revealed significant Treatment (*F*_(2,25)_ = 8.96, *P* < 0.001) and Session effects (*F*_(4,100)_ = 103.85, *P* < 0.001) and a significant Treatment × Session interaction (*F*_(8,100)_ = 5.40, *P* < 0.001).

Between-group analysis confirmed that the OXA and Fasted groups developed a stronger COA that extinguished more slowly than the aCSF group. One-way ANOVA on the mean scented water intake confirmed these observations, with inter-group differences at Day 11, Day 12, Day 13 and Day 14 (*F*_(2,25)_ = 12.86, *P* < 0.001; (*F*_(2,25)_ = 8.51, *P* < 0.01; *F*_(2,25)_ = 9.95, *P* < 0.001 and *F*_(2,25)_ = 6.83, *P* < 0.01 respectively). *Post-hoc* Bonferroni pairwise comparisons revealed significant differences between aCSF and both OXA and Fasted groups at Day 11, Day 12 and Day 13 (from *P* < 0.001 to *P* < 0.05), while the OXA group differed from the aCSF and Fasted groups at Day 14 (*P* < 0.01 and *P* < 0.05 respectively). One-way ANOVA within-group comparisons showed a significant effect of factor Session on COA performances in each of the groups (*F*_(4,40)_ = 9.99; *P* < 0.001; *F*_(4.45)_ = 12.54; *P* < 0.001 and *F*_(4,40)_ = 15.85; *P* < 0.001 for aCSF, OXA and fasted groups respectively). In the aCSF group the extinction curve increased rapidly between D11 and D15. *Post-hoc* Bonferroni’s tests confirmed this observation and showed only a significant difference in the variables obtained in D11 and D12 compared to those obtained in D14 and D15 (*P* < 0.05 to 0.001). In the OXA and Fasted group, extinction curves reached a ceiling more slowly than the aCSF group. *Post-hoc* Bonferroni tests confirmed this observation and showed a significant difference in the means solution intake in D11 and D12 compared to D14 and D15 (*P* < 0.05 to 0.001) and a significant difference in the same variable between D13 and D15 (*P* < 0.001 and *P* < 0.01) in the OXA and Fasted groups respectively).

These statistical analyses show that scented water (CS) intake increased significantly in each group throughout the sessions, but it has to be borne in mind that the curves combine two distinct if related phenomena: extinction of COA learning and extinction of CS neophobia.

Concerning the neophobia, the figure shows a decrease in the mean solution intake between D7 and D8 in the three experimental groups and the decrease appears to be stronger in the Fasted group. A two-factor ANOVA with repeated measures with Treatment (aCSF, OXA and Fasted) as between-subjects variable and Session (Day 7 versus Day 8) as within-subject variable confirmed this description and revealed significant Treatment (*F*_(2,25)_ = 6.24, *P* < 0.01) and Session effects (*F*_(1,25)_ = 395.3, *P* < 0.001) and a significant Treatment × Session interaction (*F*_(2,25)_ = 27.1, *P* < 0.001). Pairwise intergroup comparisons (one-way ANOVA) indicated no difference between groups on D7 and a significant decrease in mean scented water intake in Fasted group compared to aCSF and OXA groups (one-way ANOVA (2,25) = 28.92; *P* < 0.001, *post-hoc* Bonferroni *P* < 0.001).

Experimental COA extinction reflects not loss of the original memory trace but rather new learning whereby the CS comes to predict no US. It has been suggested that this learning inhibits the previously acquired conditioned response to the olfactory CS (Rescorla and Heth, 1975; Robbins, 1990).

Therefore, and in order to establish the real number of sessions needed to extinguish COA in each group taking the neophobia effect into account, additional analyses were performed considering the mean scented water intake measured during the acquisition in the variables.

One-way ANOVAs performed on these variables revealed a significant effect of factor Session in the aCSF and OXA groups (*F*_(5,48)_ = 8.17; *P* < 0.001; *F*_(5,54)_ = 14.1; *P* < 0.001 respectively). In the aCSF group, *post-hoc* Bonferroni analyses revealed that only mean scented water intake at Day 11 significantly differed from Day 8 (*P* < 0.01), suggesting that COA extinguished in a single session in this group. In the OXA group, *post-hoc* Bonferroni analyses revealed significant differences in the data obtained at Day 8 versus Day 11, Day 12 and Day 13 (*P* < 0.001, *P* < 0.001 and *P* < 0.05 respectively), suggesting that COA extinguished over three sessions in this group. Concerning the Fasted group, the fact that animals were conditioned and tested under two different conditions (food-deprived versus food-satiated), rendered the comparison between D8 and the following extinction sessions irrelevant due to the fact that the high level of CS neophobia displayed by the Fasted group on D8 may be attributed to stress induced by the fasting condition (see Section Discussion). Therefore, the data obtained in the Fasted group from Days 11 to 15 were compared to those of the aCSF group on Day 8. Here, one-way ANOVA revealed an effect of the session (*F*_(5,48)_ = 16.2; *P* < 0.001) and *post-hoc* Bonferroni tests revealed a significant difference between data measured at D8 versus D11, D12 and D13 (*P* < 0.001, *P* < 0.001 and *P* < 0.05 respectively), suggesting that COA extinguished over three sessions in this group.

Taken together, these data show that COA extinction was significantly faster in the aCSF than in the OXA and Fasted groups. Moreover, the fact that the OXA group differed from the aCSF and Fasted groups at Day 14 might suggest that the process of COA extinction differed between OXA and fasted groups.

## Discussion

One of the main results represented on Figure 1 was that fasting and central OXA infusion induced similar COA enhancement compared to the Control group. The fact that all animals were satiated during the test rules out the possibility that factors such as olfactory hypersensitivity or stress induced by food-deprivation may have influenced the process of COA extinction and/or retrieval.

As shown in Figure 1, the Fasted group displayed a decrease in mean scented solution intake between the last water-drinking habituation session (Day 7) and the acquisition (Day 8), suggesting that, despite the use of a very low concentration of isoamylacetate solution (ISO, 10^−6^), the acute 24-h food and water deprivation schedule enhanced neophobia for a novel olfactory stimulus.

Neural processing of olfactory information is closely linked to the physiological and nutritional status of the organism, and fasting has been shown to increase OB reactivity (Pager et al., 1972; Apelbaum et al., 2005) and olfactory detection in rats (Aimé et al., 2007). Therefore, the strong neophobia toward the scented water observed in the Fasted group during acquisition may have resulted from the enhanced olfactory sensitivity induced by starvation. Although this starvation effect on olfactory sensitivity has been suggested to be mediated by activation of the central OXA system (Julliard et al., 2007), the very slight difference observed at acquisition (Day 8) between the OXA and aCSF groups suggests that the neophobia toward the CS displayed by the Fasted group at acquisition cannot have been simply and exclusively due to OXA-induced enhancement of olfactory detection under fasting. In this respect, data have shown that short-term (24-h) food deprivation induced a significant increase in serum corticosterone (Das et al., 2005; Johansson et al., 2008; Nowland et al., 2011) and anxiety levels (Ferry and Duchamp-Viret, 2014). Therefore, it can be assumed that the strong neophobia observed in the Fasted group at COA acquisition may have resulted, at least in part, from the combination of enhanced olfactory detection and increased anxiety induced by fasting. Moreover, the fact that OXA infusion did not induce any anxiety (Ferry and Duchamp-Viret, 2014) suggests the strong neophobia observed in the Fasted group at Day 8 was unlikely to have been mediated by central release of OXA.

The results presented in Figure 1 show that COA extinction differed between groups: while the aCSF group needed one session to extinguish COA, the Fasted and OXA groups needed three. The extinction phenomenon reflects the inhibition of the conditioned response by new learning of CS-no US (Rescorla and Heth, 1975) and it has been suggested that resistance to extinction of a CS-US association is directly dependent on the strength of the CS-US memory trace (Eisenberg et al., 2003). The present results suggest that the fasting and OXA infusion conditions both enhanced CS-US association strength, and the similarity between the Fasted and OXA groups in terms of extinction suggests that the enhancing effect of fasting on CS-US association strength is mediated, at least in part, by a central release of OXA.

Now COA is a trace conditioning that results from several processes that follow one another over time: during acquisition, CS and US processing is followed by association of the two stimuli; then, the CS-US association is consolidated and finally retrieved during the test when the CS is presented for the second time. Some studies have shown that behavioral effects of icv OXA infusion, such as feeding and drinking behavior or olfactory hypersensitivity, persist for at least 3 h (Sakurai et al., 1998; Edwards et al., 1999; Kunii et al., 1999; Julliard et al., 2007). Therefore, the effects of fasting and OXA infusion on COA observed in the present study may have resulted from changes in the processes of acquisition and/or consolidation taking place on Day 8.

### Hypothesis on the processes by which fasting and OXA may have influenced COA learning

#### CS-US Acquisition

##### Olfactory hypersensitivity and olfactory memory trace duration

As discussed in the Introduction, the acquisition of COA reflects the association between the memory trace of the olfactory CS and the delayed visceral US (see Bures and Buresova, 1990; Roldan and Bures, 1994). Several studies have shown that, when ingested, a tasteless olfactory stimulus (such as ISO) acquires a strong aversive value, even with CS-US intervals equivalent to those generally used for tastes (Rusiniak et al., 1982; Bouton et al., 1986; Slotnick et al., 1997; Chapuis et al., 2007). At a concentration of 10^−4^ in tap water, ISO, used as the CS for COA, has been shown to be resistant to a relatively long interval (up to 30 min) before delivery of the US (Chapuis et al., 2007; Ferry et al., 2007; Miranda et al., 2007). Interestingly, results obtained in aCSF group showed that ISO mixed in tap water at a concentration of 10^−6^ was able to induce a mild COA that extinguished in one session when the time interval (ISI) separating the CS from the US was about 20 min. Therefore, it is suggested that, in our conditions, the CS trace decayed over the 20-min ISI, at the end of which it is weakly associated to the US.

The effectiveness of an olfactory CS in inducing strong COA when paired with a delayed illness has been shown to be directly related to the intensity of the CS used during acquisition (Rusiniak et al., 1982; Slotnick et al., 1997). Thus, in the light of the increased olfactory sensitivity previously reported with 24-h fasting and icv OXA infusion (Julliard et al., 2007), it is possible that the strong COA obtained in the Fasted and OXA groups was directly linked to an enhanced memory for the CS resulting from the change in olfactory perception. Interestingly, a similar cause-effect relationship was described in a previous experiment by our team, in which entorhinal cortex lesion enhanced COA learning performances, an effect that was accompanied by olfactory hypersensitivity (Ferry et al., 1996). Therefore, by enhancing olfactory sensitivity, fasting and OXA infusion may have enhanced the duration of the olfactory trace by enhancing CS salience, thus rendering possible its association to the delayed toxicosis.

##### Higher US processing

In the same vein, Garcia and Koelling (1967) demonstrated that the strength of a conditioned response to an ingested CS previously paired with a gastric malaise US varies directly with the intensity of the US. Concerning the COA, similar results have been described and the strength of COA varied directly with the intensity of the US used (Rusiniak et al., 1982; Bouton et al., 1986; Slotnick et al., 1997; Chapuis et al., 2007; Ferry et al., 2007). It could therefore be argued that the enhanced COA observed in the fasted group resulted from the visceral discomfort induced by injecting the LiCl used in the COA protocol, being more intense in a 24-h fasted animal, enhancing the strength of the CS-US association. However, some studies have shown that the orexinergic system is independent of the system that deals with visceral processing of the LiCl-induced intoxication in conditioned aversive learning (Touzani and Sclafani, 2002; Di Sebastiano et al., 2011). Therefore, any indirect effect of fasting or OXA infusion on COA strength via a change in US processing may be discounted.

Regardless of this, Winsky-Sommerer et al. (2004) have shown that OXA system is activated by the corticotropin-releasing factor (CRF) that is released in condition of acute stress. Considering these data, it could be suggested that the stress induced by the US administration may have influenced the release of OXA through the activation of the CRF system in both groups of animals. If the fasting condition also enhanced the OXA level by the same route, it could be argued that the similarity in the COA performances obtained in Fasted and OXA groups was due to similar enhanced level of OXA with the different experimental conditions. If so, this would suggest that the stress induced by the US LiCl may have influenced the CS-US association or the processes underlying the CS-US memory. Even though this hypothesis could explain the similarity in the COA performances between Fasted and satiated OXA groups, however future studies will aim at verifying whether an acute restraint stress can be physiologically compared to this induced by a LiCl-induced intoxication and also whether the administration of the US in our conditions can induce OXA release.

#### CS-US consolidation

Finally, the temporal evolution of CS-US consolidation remains largely unknown; however, Dudai (1996) and Dudai and Morris (2000) proposed that consolidation involves two types of processes: synaptic consolidation, accomplished within the first minutes to hours after the CS-US association has been acquired; and system consolidation, involving reorganization of the brain circuits encoding the memory, which takes weeks, months or even years to be accomplished. Considering the duration of OXA action (at least 3 h, Sakurai et al., 1998; Edwards et al., 1999; Kunii et al., 1999; Julliard et al., 2007), it may be suggested that the enhanced COA observed in the OXA group was mediated by enhanced synaptic consolidation of the memory processes. According to this view, recent data have shown that central OXA system is involved in the acquisition and in the consolidation of fear conditioned learning (Soya et al., 2013). In order to test the involvement of the OXA system in COA consolidation process, it could be of interest to test the effect of selective blockade of OXA receptors at various times during COA learning. In this way, we assume that if the OXA system is selectively involved in the CS-US acquisition process, infusion of the antagonist before the CS presentation would disrupt both short (3–4 h after acquisition) and long term (24 h) memories. In contrast, if the OXA system is involved in consolidation, the pharmacological blockade of OXA receptors would impair selectively long-term memory leaving short-term memory intact (Sears et al., 2013).

As a first conclusion, the present results show that physiological or OXA-induced fasting affected COA through changes in memory processes occurring during the acquisition of a CS-US association and/or during the synaptic consolidation of this association. The olfactory hypersensitivity induced by fasting may influence acquisition of the CS-US association by enhancing the formation and maintenance of the CS memory trace.

### Hypothesis on the neurobiological substrate involved in the effects of fasting and OXA on COA

The neurobiological substrate through which OXA affects the memory processes underlying COA learning remains to be elucidated. However, some reports open up a number of possible hypotheses according to which OXA release during fasting may enhance learning performance through a direct or indirect influence on particular hypothalamic projection targets.

#### Olfactory bulb (OB) and locus coeruleus (LC)

As previously mentioned, olfactory sensitivity cannot be dissociated from olfactory memory, and some data suggest that the indirect effects of fasting and OXA on the olfactory memory trace formation through increased olfactory sensitivity may involve the olfactory bulb (OB). As previously mentioned, olfactory sensitivity cannot be dissociated from olfactory memory, and some data suggest that the indirect effects of fasting and OXA on the olfactory memory trace formation through increased olfactory sensitivity may involve the OB. Firstly, in addition to its well documented role in detection and discrimination, the OB is involved in memory processes underlying various kinds of olfactory learning in adult rats (see Mandairon and Linster, 2009 for review). Moreover, the OB receives direct OXA innervation from the LH (de Lecea et al., 1998; Peyron et al., 1998; Sakurai et al., 1998; Shibata et al., 2008) and an increase in OB electrophysiological response induced by fasting and OXA has been described (Pager et al., 1972; Gervais and Pager, 1982; Apelbaum and Chaput, 2003; Apelbaum et al., 2005; Hardy et al., 2005). In addition, Prud’homme et al. (2009) found that OXA antagonist treatment blocked the enhancement of OB Fos responses to a food odor. Secondly, some evidence suggests that the OXA neurons terminating in the locus coeruleus (LC) may provide a second indirect pathway for orexinergic modulation of olfactory processing: direct OXA fibers innervate the LC (Horvath et al., 1999) and activation of OXA receptors in the LC increases cell firing of intrinsic noradrenergic (NA) neurons (Trivedi et al., 1998; Hagan et al., 1999). In addition, the LC projects over 40% of its neurons directly into the OB (McLean et al., 1989) and this large NA input has been shown to modulate OB excitability, olfactory perception and olfactory learning and memory abilities (see Devore and Linster, 2012 for review).

These data suggest that the fasting-induced increase in olfactory sensitivity observed in the present and other studies probably involved the OXA system in the OB. Moreover, and in the light of the work by Escanilla et al. (2012), it may be suggested that this increased olfactory sensitivity resulted directly from OXA system activation (Hardy et al., 2005) in the OB and/or indirectly through the effect of LC-OXA system activation on NA release in the OB. Finally, it is possible that activation of both systems may simultaneously influence the strength of olfactory memory through enhanced olfactory processing. In order to test this hypothesis, future studies will aim at verifying whether the presentation of a new olfactory stimulus in fasted animals can be correlated to changes in NA release in the OB.

#### Amygdala and locus coeruleus (LC)

Orexinergic innervation of the extended amygdala (including the basolateral amygdala, BLA) was clearly described by Schmitt et al. (2012). OXA administered into the LH significantly elevated cFos-immunoreactivity in the amygdala (Mullett et al., 2000) and fasting increased OXA mRNA levels in the amygdala (Lu et al., 2000). OXA applied in acute rat brain slices activated neurons in the amygdala (Bisetti et al., 2006). Moreover, the amygdala receives OB and visceral inputs (Saper and Loewy, 1980; Inui et al., 2006), and may be a nodal point at which olfactory and neuroendocrine stimuli are integrated to modulate feeding behavior (King, 2006).

Otherwise, a large amount of data indicates that the amygdala, and more precisely the BLA, is involved in the acquisition of COA (Sevelinges et al., 2009) and more precisely in the processes underlying the formation of the olfactory memory trace and its maintenance across the ISI during COA (Ferry et al., 1995; Ferry and Di Scala, 1997, 2000). Although a direct effect of starvation-induced OXA release in the amygdala on COA cannot be excluded, to our knowledge, the involvement of the OXA system in the amygdala has never been demonstrated in learning and memory.

On the other hand, some data indicate that the enhancing effect of starvation on COA memory processes could be indirectly mediated by activation of the NA system in the amygdala. The LC projects strongly onto the amygdala (Fallon et al., 1978) and the BLA β-adrenergic system is involved in the memory processes underlying the association between odor and delayed US during COA (Miranda et al., 2007). Given the direct action of LH orexinergic neurons on the LC (Horvath et al., 1999; van den Pol et al., 2002), activation of the LC-amygdala NA system during processing of the new odor CS may be potentiated by fasting-induced OXA release. Possibly, the strength of the olfactory memory trace, and/or its association to the US, was influenced by activation of this pathway in the Fasted and OXA groups.

**Figure 2:**
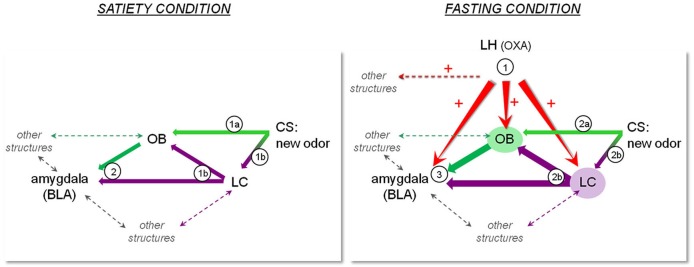
**Representation of a hypothetic model according to which OXA terminating in regions such as the OB, LC and amygdala may constitute a pathway for orexinergic modulation of the olfactory memory trace formation underlying COA**. The left panel represents the sequence of events that may take place during presentation of a new olfactory CS in the satiated condition. 1a) Olfactory CS induces activation of the OB. 1b) The novelty of the olfactory CS induces LC activation, which results in NA release in the OB and BLA. 2) The olfactory information is transmitted to the BLA where the odor trace is formed pending its association with the US. In the case of a short ISI, this sequence of events results in normal COA. The right panel represents the sequence of events that may take place during presentation of a new olfactory CS in the fasting condition. Fasting induces release of OXA in the OB, BLA and LC, preparing the system to respond to any food-odor event. 2a) Olfactory CS induces activation of the OB, potentiated by activation of the OXA system and LC-mediated NA system in the OB. In the fasting condition, CS leads to improved olfactory detection and processing. 2b) The novelty of the olfactory CS induces LC activation, resulting in enhanced NA release in the OB and BLA. 3) The enhanced olfactory information is transmitted to the BLA. Combined with OXA system activation, LC-mediated NA release in the BLA and potentiated OB activation, the olfactory memory trace strengthened or lengthened, and can thus be associated to a delayed US. This sequence of events results in COA when a long ISI is used.

Although the results shown on Figure 1 do not identify particular structures receiving OXA projections as being involved in the memory processes underlying COA acquisition, all the above-mentioned data lead us to propose a model according to which OXA release during fasting enhances learning performance through a direct or indirect influence on the circuit involved in COA. Our model shown on Figure 2 comprises some of the structures involved in this circuit including the LC, amygdala and OB, which receive LH-OXA projections. According to this model, the activation of the LH-OXA system induced by fasting reinforces the role of each structure in the circuit by enhancing the neural processes underlying attention and olfactory memory through direct and/or indirect influences.

This model is consistent with the idea of Cleland and Linster (2005) according to which the multiple feedback and feed-forward interactions between olfactory and non-olfactory areas contribute to complex processes, such as filtering and constructing olfactory representations, and compare these representations to those previously acquired in order to cue an appropriate response to relevant stimuli. The present model also supports the hypothesis that centrifugal modulatory inputs influence olfactory processing and learning mechanisms within the OB (Sullivan et al., 2000; Linster and Cleland, 2002; Yuan et al., 2003), leading us to extend these influences to other key structures involved in attention and olfactory learning and memory (Aston-Jones and Cohen, 2005; Miranda, 2012).

Of course the list of structures included in this model is not exhaustive; involvement of other feedback and feed-forward interactions between these structures and others (e.g., piriform cortex, entorhinal cortex, orbitofrontal cortex, hippocampus, etc.) will have to be considered in order to achieve a more realistic idea of the circuit actually involved in food conditioned learning (see Ferry et al., 2006, 2007; Chapuis et al., 2009; Sahay et al., 2011; Wilson and Sullivan, 2011; Chapuis et al., 2013).

## Conclusion

Feeding behavior is part of a complex integrated adaptive system, governed by the brain, in which the processing of metabolic signals reflecting the animal’s nutritional state (gastrointestinal distention, blood glucose, feeding peptides such as OXA, etc.) and of olfactory signals indicative of food determines the appropriate response to a food source. However, the differentiation between palatable and unpalatable items that conditions ingestive behavior often depends on previous experience during which the odor of the food acquired (or did not, in the case of a new odor) a hedonic valence after feeding, through CS-US associative learning. By showing that OXA system influenced the processes underlying the CS-US association, or/and consolidation of this association during COA, the present study introduces a new mechanism by which the LH-OXA system may influence the processes that enable animals to learn to select food available in the environment and to adapt their behavior to previous experience through a modulation of complex neural circuit activity. Finally, the OXA system represents a critical link between peripheral energy balance and CNS mechanisms that coordinate olfactory processing and memory, especially in the physiological state of fasting.

## Conflict of interest statement

The author declares that the research was conducted in the absence of any commercial or financial relationships that could be construed as a potential conflict of interest.
